# Evaluating School‐Based Substance Use Services: Insights From a Systematic Review

**DOI:** 10.1111/josh.70095

**Published:** 2025-12-02

**Authors:** Hannah L. Maxey, Brittany J. Daulton, Mykayla Moore, Kelsey Binion

**Affiliations:** ^1^ Bowen Center for Health Workforce Research and Policy, Department of Family Medicine Indiana University School of Medicine Indianapolis Indiana USA; ^2^ Department of Health Policy and Management Indiana University Richard M. Fairbanks School of Public Health Indianapolis Indiana USA

**Keywords:** intervention, school, screening, substance use, treatment

## Abstract

**Background:**

Substance use among youth can have lifelong consequences and therefore requires early and targeted services for those at risk. Schools possess a unique opportunity to provide substance use services to youth for both prevention and intervention. However, limited research exists on the school‐based substance use services and their effectiveness.

**Methods:**

Using PRISMA guidelines, online databases were searched for studies done between 2004 and 2024 on school‐based substance use services, their outcomes, and the characteristics of those administering them.

**Findings:**

Results showed school‐based substance use services being offered in multiple settings. Screening and intervention were the most common services provided. Although specific outcomes varied by study, including academic performance, perceptions, and actions, most were positive.

**Implications for School Health Policy, Practice, and Equity:**

Schools should create strategic plans for feasible and sustainable substance use services. Use of the screening, brief intervention, and referral to treatment (SBIRT) framework can be used to organize these efforts. Establishing robust referral networks is of particular importance for schools.

**Conclusions:**

This review highlights opportunities for schools to focus on screening and brief intervention for in‐school services while also building a strong referral network for times when treatment is necessary.

## 1 Introduction

Substance use among youth in the United States (US) is a critical public health crisis [1]. The use of substances, both illegal drugs and alcohol, place youth at a high risk for severe consequences, including impaired brain development, poor academic performance, increased risky behavior, infectious disease, accidents, injury, and death [2, 3, 4, 5, 6, 7]. Addressing youth substance use is crucial for a healthier future, as many adults with substance use problems began using as adolescents [8].

Substance use among youth in the US has decreased since 2017, but drug‐related fatalities have generally increased in recent years. Among persons aged 14–18 years, overdose deaths increased 94% from 2019 to 2020 and 20% from 2020 to 2021 [9]. This trend changed in 2022, where overdose deaths began to decrease and were continuing to do so through 2024 [10, 11, 12, 13]. This suggests that fewer youth are using substances, but those who do may face higher risks [9, 10, 11, 12]. Despite a decrease in overall substance use among youth, the consequences of use remain a challenge. In 2023, 22% of high school students reported alcohol use, 10% had used illicit drugs, and 12% had misused prescription opioids [14]. Substance use during youth has long‐term impacts on health risks [15, 16]. Recent data suggests that nearly three‐quarters of adults aged 18–30 in treatment centers began using substances at the age of 17 or younger [15, 17].

Substance use among youth and potential lifelong consequences necessitate early and targeted services for those at risk. Substance use services for youth can be categorized into three areas: screening, intervention, and treatment. Screening involves assessing individual risk of substance use to determine whether additional services are needed. Interventions provide structured feedback to encourage self‐reflection and motivate changes in substance use behaviors. Treatment involves delivery of medical care services for substance use disorder or related conditions. Reaching youth with these services is essential to reducing the burden and consequences of substance use [18]. SBIRT, a framework which provides screening, brief intervention, and referral to treatment, is a suite of services. The public health focused framework was developed in the 1980's by the Substance Abuse and Mental Health Services Administration (SAMHSA) and the World Health Organization (WHO), to address substance use [19]. Although not developed specifically for school‐based use, it has been utilized and tested across a wide range of age groups (including youth) and settings [20]. SBIRT incorporates a universal screening process using a validated screening tool. Based on the screen's outcomes, there are then prompts of positive reinforcement, a brief motivational intervention or treatment, or a referral for further assessment via residential or outpatient treatment services. SBIRT aims to support prevention, early intervention, and treatment gaps by identifying substance use risk early and integrating an appropriate response into routine health care or school programming [21]. Schools provide a unique opportunity to reach a broad population of youth, making them ideal for delivering services to reduce substance use and support students in person‐centered recovery [22, 23, 24]. The US education system is largely comprised of traditional (categorized as regular) schools and alternative schools as reported in 2023 [25]. Traditional schools serve most students, with more than 90,000 sites [26]. Alternative schools cater to students whose educational, social, and behavioral needs are typically not met by traditional schools. They are the second largest category, with over 5000 sites [25]. As of 2021, there were also 42 specialized recovery high schools that provide services for youth in recovery from substance use disorder or co‐occurring disorders. Some schools have additional capacity for services through health care partnerships with school‐based health centers (SBHCs). SBHCs are health care facilities collocated within schools that increase access to licensed health care professionals who can screen, intervene, or treat substance use, among other health conditions [27]. As of 2022, there were 3900 SBHCs in operation across the US [27].

School‐based substance use services hold the promise of increased access for youth. The capacity to provide substance use services likely varies between schools and by type of school based on available resources, such as funding, staffing, and health care partnerships. Early initiatives and research related to substance use within schools addressed universal prevention programming, which is provided to entire student bodies or large groups of students [28, 29, 30]. Few universal programs were found to be effective at changing substance use, so research called for expansion of targeted early interventions to reduce use among youth [28, 29, 30]. Evidence suggests that individually focused interventions delivered in schools may be more effective at reducing risk of substance use [28, 29, 30]. Unlike universal programming, screening, interventions, and treatment for substance use typically require some level of specialized knowledge and skills. In some instances, professional licenses or certifications are needed.

Universal programs have been extensively tested and adopted, with literature detailing their design, the personnel delivering the programs, and associated outcomes [28, 29, 30]. Conversely, there is limited research on the implementation of more targeted substance use services in schools and their outcomes. To address this gap, this study conducted a systematic literature review to identify the types of substance use services provided for youth in school settings, the workforce delivering these services, and the associated outcomes.

## 2 Methods

**FIGURE 1:**
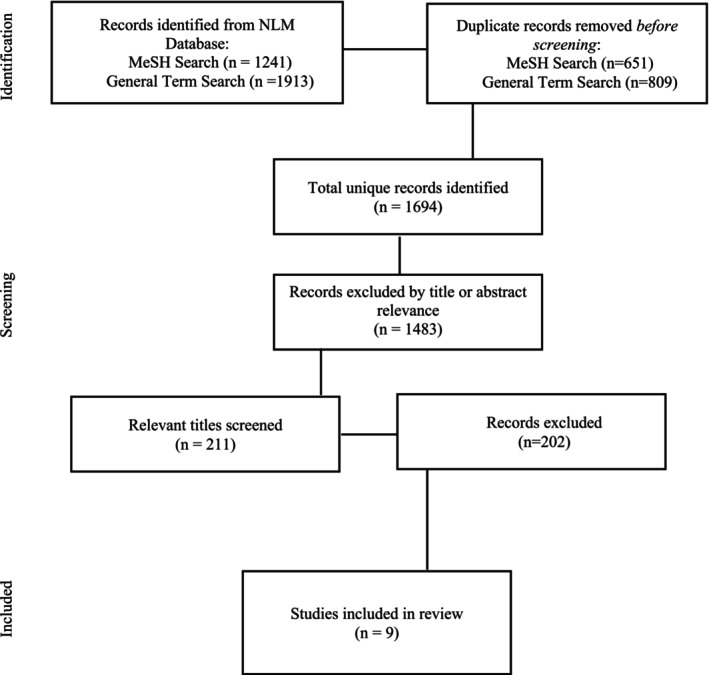
PRISMA flow chart.

The Preferred Reporting Items for Systematic Reviews and Meta‐Analyses (PRISMA) checklist informed the methodology (Figure 1) [31].

### 2.1 Identification and Selection of Studies

Prior to the literature searches, relevant search terms were selected using the National Library of Medicine (NLM) MeSH term database. MeSH (Table 1) and general term (Table 2) searches were conducted using the NLM database. The NLM database is frequently used for systematic reviews and was selected because they offer comprehensive coverage of biomedical and life sciences literature and rigorous indexing [32]. Literature from relevant organizations (e.g., Substance Abuse and Mental Health Services Administration, Centers for Medicare and Medicaid Services) was also reviewed.

**TABLE 1:** Search queries of MeSH terms (9).

MeSH term searches
(((“Substance‐Related Disorders/prevention and control”[Mesh] OR “Substance‐Related Disorders/psychology”[Mesh] OR “Substance‐Related Disorders/rehabilitation”[Mesh] OR “Substance‐Related Disorders/therapy”[Mesh])) AND “School Health Services”[Mesh]) AND “Health Workforce”[Mesh]
((“Substance‐Related Disorders”[Mesh]) AND “Adolescent”[Mesh]) AND “School Health Services”[Mesh]
((“Substance‐Related Disorders”[Mesh]) AND “Health Workforce”[Mesh]) AND “Schools”[Mesh]
((“Substance‐Related Disorders”[Mesh]) AND “Health Workforce”[Mesh]) AND “School Health Services”[Mesh]
((“Substance‐Related Disorders”[Mesh]) AND “School Health Services”[Mesh]) AND “Students”[Mesh]
((“Delivery of Health Care”[Mesh]) AND “Substance‐Related Disorders”[Mesh]) AND “School Health Services”[Mesh]
((“Substance‐Related Disorders”[Mesh]) AND “School Teachers”[Mesh]) AND “School Health Services”[Mesh]
(((“Adolescent”[Mesh]) AND “School Health Services”[Mesh]) AND “Substance‐Related Disorders”[Mesh]) AND “Health Workforce”[Mesh]
(“Substance‐Related Disorders”[Mesh]) AND “School Health Services”[Mesh]

**TABLE 2:** Search queries of general terms (37).

General term searches
“substance use disorder” and “school‐based services”
“substance use disorder” and “services” and “youth” and “school”
“substance use disorder” and “school‐based treatment”
“substance use disorder” and “alternative school”
“substance use disorder services” and “school”
substance use disorder services and “traditional” and “school”
“substance use disorder services” and “youth” and “school”
“substance use disorder” and “services” and “school‐based”
“substance use disorder” and “services” and “school” and “youth”
“substance use disorder” and “services” and “school” and “professionals”
“alternative high school” and “substance use” and “services”
recovery high school and “substance use” and “services”
non‐traditional high school and “substance use” and “services”
“substance use” and “services” and “high school”
“substance use” and “services” and “middle school”
“substance use” and “services” and “elementary school”
“substance use” and “recovery high school”
“substance use disorder” and “adolescent” and “treatment” and “school”
“substance use disorder services” and “school”
“substance use disorder treatment” and “high school”
“substance use disorder treatment” and “recovery high school”
“substance use disorder treatment” and “non‐traditional high school”
“substance use disorder” and “service” and “high school”
“substance use disorder” and “service” and “middle school”
“substance use disorder” and “service” and “elementary school”
“substance use disorder” and “alternative” and “school”
“health workforce” and “school health services” and “substance use disorder”
“health workforce” and “school‐based services” and “substance use disorder”
“health workforce” and “students” and “substance use disorder”
“workforce” and “substance use” and “school”
health workforce and substance use services in high schools
“alternative high school”
“substance use” and “high school” and “prevention” and “outcomes”
“school‐based” and “substance use” and “prevention”
“school‐based” and “substance use” and “services”
“school‐based” and “substance use” and “treatment”
“school‐based” and “substance use” and “personnel”

Searches were filtered for work published between 2004 and 2024, so only the most recent research would be included. Results were limited to include human subjects and the English language.

Three researchers managed the article review process. The first researcher conducted the literature searches in the NLM database. The searches yielded 3154 hits, of which 1460 were duplicates and removed prior to screening of titles and abstracts. The first researcher then performed a systematic screening of titles and abstracts on the 1694 unique articles and identified 211 for full review. Two additional researchers performed a comprehensive review of the 211 articles using inclusion criteria established for this study. Articles had to meet all the following criteria to be included: (1) school‐based (either in traditional, alternative, or recovery schools or SBHCs) substance use services (screening, intervention, or treatment) were provided; (2) workforce characteristics of those providing school‐based substance use services were described; and (3) substance use service outcomes (action, perception, or academic) were measured. The authors excluded studies on universal programs delivered to entire student populations regardless of risk, although universal screenings were included. Universal programs can prevent abuse or misuse, but they do not target a specific population and are thus not considered services [33]. Universal screenings, however, allow for identification of individuals in need of intervention, referral, or treatment. Nine (*n* = 9) studies were retained for synthesis (Table 3).

**TABLE 3:** Literature included in systematic literature review.

Authors	Title	Screening	Intervention	Treatment	Type of school (traditional/ alternative/ recovery)	SBHC (Y/N)	Study conditions	Specific outcomes/ results	Outcomes classification (action/ perception/ academic)	Workforce characteristics
Gryczynski et al. [34]	Computer versus. Nurse Practitioner Delivered Brief Intervention for Adolescent Marijuana, Alcohol, and Sex Risk Behaviors in School‐Based Health Centers	CRAFFT (self‐report)	MI		Traditional (public)	Y	Computer‐based intervention versus nurse practitioner at an SBHC‐delivered intervention	Intervention led to lower frequency of marijuana and alcohol use and alcohol‐specific problems compared to assessment‐only students, regardless of condition. No difference in behavioral outcomes between conditions. High satisfaction was reported in both conditions.	Action and perception	Screening provided by research assistants Intervention was delivered via (1) computer or (2) nurse practitioner
Chadi et al. [35]	Student Experience of School Screening, Brief Intervention, and Referral to Treatment (SBIRT)	CRAFFT (self‐report)	MI		Traditional (public)	N	Survey of students who received SBIRT at school in Massachusetts (all received intervention)	Most students indicated that the information received during SBIRT was useful. Speaking to an adult about drugs or alcohol was valuable. Students also report they would be comfortable returning to the person who screened them with questions about drugs and/or alcohol in the future.	Perception	Screening and intervention provided by school staff either (1) nurse, (2) psychologist, or (3) guidance counselor
Lintz et al. [36]	Associations Between School‐Based Substance Use Treatment and Academic Outcomes	Clinical interview (self‐report)	MI	Acceptance and commitment therapy, family sessions, case management, psychiatric consultation with medication management (prescribing psychotropics)	Traditional (public)	Y	Secondary review of data from participants who were enrolled in a 23‐week program of care for substance use disorder at SBHC in Colorado	49% of participants achieved a negative urine drug screen; a decrease in the number of classes missed; reduction in behavioral incidents. There was not a significant change in GPA.	Action and academic	Screening provided by LCSW or board‐certified child psychiatrist Intervention provided by LCSW Treatment was provided by board‐certified child psychiatrist; school‐based therapists
McCarty et al. [37]	Screening and Brief Intervention with Adolescents with Risky Alcohol Use in School‐Based Health Centers: A Randomized Clinical Trial of the Check Yourself Tool	Check yourself tool and CRAFFT (self‐report)	Check yourself feedback tool and an SBHC visit		Traditional and alternative (2 large urban high schools and 1 small alternative high school)	Y	Randomized control trial of SBHC visit with the Check Yourself tool feedback or just a regular SBHC visit	Both groups decreased the number of drinks, maximum number of drinks, and time high on marijuana over time (at 2‐month follow‐up).	Action	Screening provided by a tablet prior to appointment at SBHC Intervention was provided by health care providers (either advanced practice registered nurse or mental health counselor) at the SBHC visit; Check Yourself tool was provided by computer
Levy et al. [38]	Association of Screening and Brief Intervention with Substance Use in Massachusetts Middle and High Schools	CRAFFT (self‐report)	Providing brief advice about the risks of alcohol and other drug use or positive reinforcement of positive alcohol and/or drug behaviors		Traditional	N	Post‐intervention of required SBIRT for students in schools; quality improvement study. Compares middle school and high school students; male and female	Students in a school grade exposed to SBI reported smaller increases in the rate of cannabis use compared with students who did not receive SBI. Cannabis use increased in both groups, as well as e‐cigarette use. SBI was most successful for middle school students. Smaller increases in use reported for both middle school and female students.	Action	Screening and intervention provided by school staff members trained in SBIRT, exact roles and titles not mentioned
Stormshak et al. [39]	An Ecological Approach to Promoting Early Adolescent Mental Health and Social Adaptation: Family‐Centered Intervention in Public Middle Schools		FRC offering family check‐Up (FCU) utilizing MI		Traditional	N	Intervention trial comparing FCU intervention to FRC services as usual	Intervention effects for all four outcomes were found, indicating that FCU was successful at reducing the antisocial behavior and use of alcohol, tobacco, and marijuana among middle school youth whose families engaged with treatment.	Action	Intervention provided by a “parent consultant,” individuals trained in the intervention to directly work with parents of the adolescents through their roles at the school's FRC
Mitchell et al. [40]	Screening, Brief Intervention, and Referral to Treatment (SBIRT) for Substance Use in a School‐based Program: Services and Outcomes	CRAFFT (self‐report)	MI	ACRA (adolescent community reinforcement approach)	Traditional	Y	Clinical intervention looking at differences between those receiving brief intervention (BI), brief treatment (BT), or referral to treatment (RT)	Those receiving SBIRT services, regardless of condition or intensity, reported significant reductions in days of drinking to intoxication and drug use. No significant difference in consuming alcohol.	Action	Screening and intervention done in SBHC; all master's‐level behavioral health counselors
Clark et al. [37]	Project Success' Effects on Substance Use‐Related Attitudes and Behaviors: A Randomized Controlled Trial in Alternative High Schools	77‐item self‐report questionnaire (self‐report)	Project SUCCESS (schools using coordinated community efforts to strengthen students)		Alternative	N	Randomized control trial measuring effectiveness of intervention (Washington state), intervention versus no intervention	Both study conditions showed increases in perceptions of harm from alcohol use, alcohol and marijuana resistance self‐efficacy, peer support, rebelliousness, and peer pressure to use alcohol and marijuana. More harm was noted for the intervention group, but peer support increased more in the control group.	Perception	Screening and intervention provided by master's‐level professional counselors in the schools
Winters et al. [41]	Brief Intervention for drug‐abusing adolescents in a school setting: Outcomes and Mediating Factors	Adolescent diagnostic interview, timeline follow back, personal consequences scale, stages of change, problem‐solving questionnaire (self‐report)	MI		Traditional	N	Intervention trial comparing a brief intervention with adolescents only, a brief intervention with the adolescent and parent, or assessment only	Both active conditions showed significant improvements across outcomes compared with the assessment‐only group (alcohol use days, cannabis use days, alcohol abuse symptoms, alcohol symptoms, and drug use consequences). The group that included a parent session exhibited greater and more consistent intervention effects compared with the condition in which only the adolescent client received services.	Action and Perception	Screening was provided by research assistants Intervention was provided by school counselors and/or therapists

### 2.2 Data Extraction and Analysis

Relevant information was systematically extracted from each identified article. This included citation details (author, year, title); type of school (traditional, alternative, recovery); school‐based health center status; details of substance use services (screening tools, intervention type, and/or treatment protocols); study condition (design, description of outcomes/results); and workforce characteristics (credentials and/or job titles). Following extraction, authors analyzed outcomes from the articles and developed Outcomes Classifications based on commonalities in operational definitions. Action was defined as behavior change (e.g., changes in frequency of use). Perceptions were defined as attitudes towards services and/or substanceuse (e.g., satisfaction with services, harm and/or risk of use, self‐efficacy of resistance of use, and/or normative beliefs about use). Academic outcomes were based on specific academic indicators (e.g., grade point average, school attendance, suspension, expulsion, etc.).

## 3 Results

Of the nine included studies, six described screening and intervention services [34, 35, 37, 38, 41, 42], one described intervention services only [39], and two described all three types of services [36, 40]. Four articles cited that the services were provided within the screening, brief intervention, and referral to treatment (SBIRT) framework [35, 38, 40, 42].

As far as screening was concerned, most articles (*n* = 8) described some type of screening service [34, 35, 36, 37, 38, 40, 41, 42]. Six of the studies used questionnaires only [34, 35, 37, 38, 40, 42], one used a clinical interview only [36], and one study used both types of screening [41]. Five studies reported using the CRAFFT screening tool (Car, Relax, Alone, Forget, Friends, Trouble) [34, 35, 38, 40, 42]. Other screening tools included were the Check Yourself Tool and a 77‐item self‐report questionnaire [37, 42]. Screenings were conducted by research assistants [34, 41], parent consultants employed by school family resource centers [39], licensed clinical social workers (LCSWs) or mental health counselors [36, 37, 40], or board‐certified child psychiatrists [36]. In one article, screening tools were administered by a computer‐based program [42].

All studies included reported some form of intervention. Typically, the interventions were brief (5–30 min), structured interactions conducted by a trained individual focused on reinforcing self‐determination to reduce risky behavior, providing structured feedback, and/or motivating change [43]. Most commonly, some form of interview or feedback was utilized alone (*n* = 7) [34, 35, 36, 38, 39, 40, 41]. However, one intervention included an interview and an SBHC visit (*n* = 1) [42], and another intervention was described as a community engagement strategy (*n* = 1) [37]. Motivational interviewing (MI) was the most reported strategy used for interventions in the studies, with six articles implementing the strategy [34, 35, 36, 39, 40, 41]. MI is an evidence‐based approach to behavior change that focuses on collaborative communication to help clients meet their goals by considering their own motivations [44]. Other interventions included school‐specific community engagement programs (e.g., Project Success) [37]. Interventions were most often provided by licensed health professionals, such as nurse practitioners, LCSWs, advanced practice nurses, or mental health counselors, but were also provided by school staff and other roles in some articles [34, 35, 36, 37, 40, 41, 42]. In one article, the intervention was a computer‐based MI feedback tool that gave feedback to the participant as they responded to questions, either providing the dangers of the behavior or positive reinforcement [34].

Only two articles discussed treatment in a school‐based setting, both of which provided treatment in a SBHC [36, 40]. Treatment offerings could be categorized as therapeutic approaches and community reinforcement. One study incorporated comprehensive treatment, including therapy and medication management [36]. The other study focused on brief treatment, specifically the Adolescent Community Reinforcement Approach, which aims to promote abstinence, reinforce participation in pro‐social activities, foster positive peer relationships, and enhance family relationships [40]. All treatment services were provided by licensed health or school professionals, such as board‐certified child psychiatrists, school‐based therapists or counselors, or master's‐level‐trained behavioral health counselors.

Where the workforce was concerned, screenings were the service type most often delivered by school staff, as well as interventions in some articles [35, 37, 38, 39, 41]. The articles included descriptions of school staff but were not always specific about their training or roles. For example, one article describes the use of family resource centers (FRCs) as a means of providing substance use intervention services while engaging the family [39]. Although it was clear that the FRC staff were school employees, their roles or titles were not specified. Two other articles described school staff providing screening or interventions, including school counselors, school nurses, or school psychologists. If treatment services were offered, these were always conducted by licensed health professionals (e.g., child psychiatrists or behavioral/mental health counselors) [36, 41].

The nine studies offer evidence that substance use services are provided in a variety of school settings, such as traditional and alternative schools and SBHCs. Most often, services were delivered in a traditional school setting (*n* = 7) [34, 35, 36, 38, 39, 40, 41]. One article focused on alternative schools [37, 42]. None of the literature discussed recovery high schools, nor did it provide comparisons of programs or outcomes across school settings. Regardless of school type, four studies defined their school‐based setting as SBHCs [34, 36, 40, 42]. Five studies were not in SBHCs [35, 37, 38, 39, 41].

The nine identified studies measured outcomes following the provision of substance use services in schools which authors categorized as action, perception, and/or academic. Four of the included studies measured action outcomes only [38, 39, 40, 42], two measured perceptions alone [35, 37], two measured action and perception together [34, 41], and one measured action and academic outcomes [36]. The outcomes were most often measured through self‐report, with eight of the included studies utilizing verbal or written self‐reports [34, 35, 37, 38, 39, 40, 41, 42]. Only one study used a biological marker (urine screen) to operationalize use [36]. However, it is important to note that this study included treatment as a service, with drug urine screens included in treatment protocol. Additionally, five of the included articles were studies, whether randomized control or clinical interventions, measuring the effect of services on outcomes for different groups [34, 37, 39, 41, 42]. The other four were evaluation or quality improvement studies where outcomes were measured pre/post services [35, 36, 38, 40].

Generally, school‐based services produced favorable action outcomes, typically lowering self‐reported frequency of use [34, 38, 39, 40, 41, 42]. Screenings paired with interventions were typically more successful than screenings alone, in studies where comparative analyses were conducted [34, 35, 37, 38, 41, 42]. The services also succeeded in improving perceptions, such as increased satisfaction with services, increased understanding of harm and risk, and increased self‐efficacy to resist use [34, 35, 37, 41]. One study found that substance use services (screening, intervention, and/or treatment) resulted in reduced negative academic outcomes (e.g., missed classes, school suspension, etc.) [36].

## 4 Discussion

This systematic literature review highlights the use of substance use services in schools, as well as the potential for positive impacts on substance use outcomes for students. However, the limited number of studies detailing specific services and the workforce involved indicates that these services are not widely or uniformly available. To address this gap, a scalable framework is essential to support the expansion of critical substance use services for youth in schools, accommodating varying resources and staff capacities.

SBIRT is a widely accepted framework for organizing and providing multiple substance use services [21]. The framework includes screening to identify risk and use status, brief intervention to provide feedback and motivation to change, and referral to treatment for those with risky substance use behavior or substance use disorder [21]. Each component of the SBIRT framework can be customized, creating flexibility in implementation across various settings. It has been widely recommended in health care provision for youth and is increasingly found in school‐based settings [45]. The SBIRT framework was specifically cited in four articles in the current review and provides a useful lens for discussing school‐based substance use services [35, 38, 40, 42].

Screening is the first component of the SBIRT framework. Substance use screenings are crucial for youth because they enable early detection of substance use, which is vital for preventing the development of misuse or disorders. The American Academy of Pediatrics recommends that regular screenings start as early as age 12 to ensure timely intervention and support [46, 47]. In the literature reviewed, CRAFFT was the most frequently cited validated screening tool. It is also recommended by the American Academy of Pediatrics for use in screening of substance use, substance use risk, and substance use disorder among youth aged 12–21 years [48]. The clinician‐ and self‐administered versions of CRAFFT are publicly available at no cost and are therefore a good fit for school‐based screening initiatives. Screenings, such as CRAFFT or other validated tools, aid in prevention by identifying early signs or risks of substance use, allowing for timely intervention before the behavior escalates.

Brief interventions are the second component of the SBIRT framework and were included as a substance use service in every study identified in this review. MI was the most reported form of brief intervention [34, 35, 36, 38, 39, 40, 41, 42]. MI is a valid intervention for behavioral change that is grounded in several widely accepted psychological models (Theory of Planned Behavior, Theory of Reasoned Action, Transtheoretical Model of Change). The success of MI as a tool for behavior change is demonstrated across a large body of clinical studies [49, 50, 51, 52, 53, 54, 55]. For example, in the smoking cessation literature, MI has been demonstrated as a successful intervention to quit smoking by increasing knowledge and improving motivation [56, 57, 58]. Additionally, MI is a very accessible intervention technique as it does not require extensive education or occupational licensure in behavioral health or counseling. Although MI is typically used by health care professionals, its components and skills can be taught to school staff, peers, and others through continuing education workshops or courses, offered both in person and online over varying lengths of time [34, 35, 59, 60]. Many individuals in various roles across school settings can be trained in MI techniques. MI should be seen as a best practice for substance use‐focused interventions within school‐based settings to best meet the needs of students.

Screening and brief intervention are seen as particularly effective for the prevention of substance use among youth, equipping students with information and feedback during screening and brief intervention to increase their understanding and assessments of harm and risk to better inform their personal decisions. However, for youth who are presenting with risky substance use behavior or with substance use disorder, treatment may be required to promote behavior change. Referral to treatment is the final component of the SBIRT framework. It does not include the provision of treatment; rather the framework ends with connecting individuals through referral to a source of care where they might receive treatment. The findings of this review suggest that referral to treatment might generally be an appropriate end goal for school‐based services, as only two studies identified in this review explored treatment services within a school‐based setting [36, 40].

Substance use treatment services require highly skilled, licensed health care and behavioral health professionals who are not traditionally employed by or practicing within school‐based settings. Although treatment has not been widely available in schools, recovery high schools and SBHCs are outliers. Recovery high schools are resourced and staffed to provide a specialized approach to education for youth with substance use disorders, where students receive treatment and complete diploma requirements simultaneously. Meanwhile, SBHCs collocate health care professionals and services within schools, which might be leveraged to increase access to substance‐use services [42, 61]. Unfortunately, recovery high schools and SBHCs are rare commodities with limited capacities [62, 63, 64, 65]. It is unreasonable to assume that these resources alone can serve the current treatment needs among youth. Expansion of treatment within school‐based settings would require significant resources and is not likely to be a feasible mechanism to meet the needs of youth. What is more feasible, and where schools should focus their attention, is the development and promotion of robust referral to treatment networks. Such networks are key to successfully connecting youth to treatment options.

Referral to treatment networks should include various types of treatment programs, such as in‐patient, outpatient, community‐based, psychiatric counseling, and so forth to ensure the diverse treatment needs of youth with substance use can be met. A network would enable school counselors or other staff involved in screenings and brief interventions to directly refer students to treatment services within their community.

National and state networks already exist within health care to support youth access to psychiatric care and may serve as an exemplary model or resource for school‐based initiatives. The National Network of Child Psychiatry Access Program (NNCPAP) is an example of a network that currently exists to support psychiatric access for youth through telephonic consultation for primary care providers to support diagnosis, treatment planning, and referral. The NNCPAP started with a state pilot and then received federal funding from the Health Resources and Services Administration for national expansion [66, 67]. Additionally, all states have a designated office or agency responsible for overseeing substance use and mental health treatment programs [68]. These entities manage the state's substance use disorder services programs and can partner with schools seeking to build a referral to treatment network.

The potential consequences of substance use among youth in the US underscore the urgent need for school‐based substance use initiatives to effectively connect at‐risk youth and those with substance use disorders to treatment. Prioritizing the development of robust referral‐to‐treatment networks is essential for any efforts to develop or expand school‐based substance use services.

## 5 Implications for School Health Policy, Practice, and Equity

This review suggests the need for comprehensive school health policies to expand and sustain substance use services, ensuring they are widely available and effective. By focusing on strategic planning and utilizing the SBIRT framework, schools can build or strengthen their capacity to provide substance use services, ultimately promoting positive behavior change and addressing substance use disorders among students.

Both prevention and treatment are essential for addressing substance use disorders and promoting behavior change. Schools should prioritize the implementation of the SBIRT framework, utilizing tools like CRAFFT for screening and MI for brief interventions. Training multiple staff members across various roles to implement these tools can enhance the effectiveness of school‐based substance use services.

Although recovery high schools and SBHCs have provided solutions in some communities, they are not widely available. Schools should focus on the referral to treatment element of SBIRT by developing robust referral networks and partnerships with clinical organizations to make treatment more accessible. These networks would enable school counselors and staff involved in screenings and brief interventions to directly refer students to appropriate treatment providers, facilitating the potential for positive changes in risky substance use behaviors.

State policymakers also play an important role in school health policies that support the expansion of substance use services. In Massachusetts, legislation passed in 2016 mandating SBIRT screening in all schools [35, 38]. This legislation was linked to an appropriation for the Department of Elementary and Secondary Education, requiring screening for all students in two grade levels, regardless of qualifying factors. The legislation requires that one middle school and one high school grade be selected for screening, and grade selection be based on local data. Unless local data suggests otherwise, statewide initiatives suggest intervention occur in Grades 7 and 9 [69]. In Colorado, SBHCs receive funding through an appropriation from the state's Department of Public Health [36]. These funds, along with contributions from foundations, community fundraising, school bonds, and applicable federal sources (e.g., American Rescue Plan Act), have been used to ensure SBHCs in Denver can provide substance use services to all students free of charge [70].

Since the COVID‐19 pandemic, 20 U.S. states have allocated funding to support school‐based mental health services [71]. More research is needed to evaluate these funding approaches for school‐based services, particularly their applicability to substance use services, as well as their sustainability and scalability. Providing substance use services requires significant capital investment, and state policymakers and legislators can support schools in the provision and expansion of these services.

Although not explicitly discussed in the articles identified in this literature review, state Medicaid programs can provide reimbursement for medically necessary substance use services delivered in school‐based settings [72]. As of 2023, 25 states have taken significant steps to expand their school Medicaid programs, thereby increasing the availability of these essential services [73]. However, the specific school‐based services covered by Medicaid, as well as the qualifications of the school health professionals eligible for reimbursement, can vary significantly from state to state [74, 75].

To navigate these variations, schools should actively collaborate with their state Medicaid programs to ensure they are fully aware of the reimbursement opportunities available for substance use services. This includes identifying which school health professionals, such as counselors, nurses, or social workers, are eligible for reimbursement under their state's Medicaid policies [75]. By doing so, schools can maximize the potential for Medicaid reimbursement, thereby enhancing their capacity to provide comprehensive substance use services to students.

Furthermore, schools should seek to align relevant state policies between education and Medicaid, particularly concerning provider qualifications and credentialing [74]. This alignment is crucial for ensuring that the services provided meet the necessary standards for Medicaid reimbursement. In instances where Medicaid reimbursement is not currently available, schools should proactively engage with state policymakers to explore opportunities for expanding Medicaid coverage to include these vital services. This may involve advocating for policy changes or additional funding, such as alternative payment models [76], to support the inclusion of substance use services in school‐based Medicaid programs.

Although these funding mechanisms offer promise, the reality is that many schools still struggle to secure sufficient resources for substance use services amid competing priorities and increasing budget constraints. Success in expanding substance use services will depend on political commitment and innovative funding approaches that recognize schools as partners in addressing the substance use crisis among youth.

## 6 Limitations

Searches conducted and filters used could be limiting, potentially not identifying every relevant study. The searches potentially excluded relevant articles if the terms were not found in the title or abstract. Additionally, due to the heterogeneity of the research, meta‐analysis was not used. A meta‐analysis would be inappropriate because the data did not meet statistical assumptions.

Several inconsistencies were reported in the literature. Researchers used various measures and definitions in their research, which created difficulties in comparing results across studies. Inconsistencies are also present in the types of interventions that were used and tested. Some interventions were case or pilot study programs that were not yet sustained, and others were state‐funded approaches to early intervention for substance use in school‐based settings. These differing approaches could lead to inconsistencies in the conclusions drawn. Additionally, it is important to note that schools may be offering substance use services that are not captured in the peer‐reviewed literature.

## 7 Conclusions

This systematic literature review offers insight into the opportunities that policymakers, education officials, and other key stakeholders should consider when deciding how to best implement and support substance use services in school‐based settings. The identified studies suggest that the SBIRT framework may potentially be a successful model for substance use services, as it has been adopted by some states and is recommended by the American Academy of Pediatrics [35, 38, 45].

Although screening, brief intervention, and referral to treatment can be helpful in reducing youth substance use (CITE), there are situations where more intensive treatment is necessary. If a student is high risk or already using substances, it is imperative that schools have the tools and resources to intervene appropriately. Early intervention is key, but treatment is commonly missing from school settings. Although treatment requires licensed professionals, often not employed by schools, schools and school systems can focus on providing screening and brief interventions with trained school staff, while creating broad partner networks for referrals to easily provide treatment options to those identified as in need. Given the alarming rate of use and potential long‐term impact of substance use and abuse, schools and health policymakers must prioritize solutions for school‐based substance use services focused on both prevention and treatment.

## Funding

This work was supported by Pew Charitable Trusts, Grant Contract ID#37025. The views expressed herein are those of the authors and do not necessarily reflect the views of The Pew Charitable Trusts.

## Ethics Statement

The authors have nothing to report.

## Conflicts of Interest

The authors declare no conflicts of interest.

## Data Availability

Data sharing not applicable to this article as no datasets were generated or analysed during the current study.
